# Neurological Sequelae After Acute Carbon Monoxide Poisoning

**DOI:** 10.7759/cureus.52840

**Published:** 2024-01-24

**Authors:** Bhushan Sudhakar Wankhade, Wasim Shabbir Shaikh, Zeyad Faoor Alrais, Adel Elsaid ElKhouly, Ammar Ali Salman

**Affiliations:** 1 Critical Care Medicine, Rashid Hospital, Dubai Academic Health Corporation, Dubai, ARE

**Keywords:** parkinsonian symptoms, hyperbaric oxygen therapy, delayed neurological sequelae, carbon monoxide poisoning, burns

## Abstract

Carbon monoxide poisoning (COP) is a common cause of death due to poisoning. After COP, a significant number of patients may develop a distinct type of neurological dysfunction called delayed neurological sequel (DNS). Recently, we came across a disaster of COP cases after a fire in a shared accommodation. The hostel was overcrowded and had a faulty air-conditioning/exhaust system. A total of five patients with loss of consciousness and shock were brought to us. They were diagnosed with acute COP based on their history of exposure to carbon monoxide (CO) and elevated carboxyhemoglobin levels in blood gas measurements. All patients were intubated and mechanically ventilated. Standard intensive care management was given to them, which included oxygenation, sedation, fluid resuscitation, and vasopressors. Their carboxyhemoglobin was rapidly reversed with normobaric oxygen therapy (NBO_2_). Three patients showed good response and neurological recovery after NBO_2_._ _Unfortunately, two patients developed DNS. DNS is a neuropsychological condition that may have cognitive, psychiatric, vestibulocochlear, motor, sensory, or diffuse demyelinating effects after COP. DNS is diagnosed in patients with a typical history of exposure to CO and a constellation of signs and symptoms. Neuroimaging, specifically magnetic resonance imaging of the brain with gadolinium contrast, is the method of choice for diagnosis. Treatment of DNS after COP begins with anticipation. All patients should receive appropriate oxygen therapy to bring down carboxyhemoglobin as soon as possible. Hyperbaric oxygen therapy (HBO_2_) for the treatment of COP and prevention of DNS is still debatable. In the available medical literature, there are conflicting recommendations regarding the use of HBO_2_ in COP/DNS. Moreover, apart from a lack of consensus, there is also a lack of clarity about optimum timing, duration, atmospheric pressure, and number of sessions of HBO_2_ in preventing DNS after COP. The development of DNS after COP is not directly responsible for mortality, but recovery sometimes takes a long time, which can contribute to increased morbidity and costs of treatment.

## Introduction

Carbon monoxide poisoning (COP) is one of the most common causes of death due to poisoning in the world. Western data suggest that COP accounts for more than 50,000 emergency cases per year in the United States [1]. About 30% of victims of burn accidents get exposed to high carbon monoxide (CO), and they can exhibit manifestations of COP or die due to it [2]. There has been a decline in mortality caused by COP because of recent improvements in the overall fire safety/evacuation protocols and intensive care unit (ICU) management [3]. Many patients after COP develop delayed neurological sequelae (DNS). DNS recovers with time, and it does not lead directly to patient mortality. However, recovery can take a prolonged time, which results in increased patient morbidity and cost of treatment. Hyperbaric oxygen therapy (HBO_2_) for the treatment of COP and prevention of DNS is still controversial. Some authors suggest a bundled approach instead of HBO_2_ alone [4]. This is especially important in situations where HBO_2_ is not radially available. We report two cases of DNS from recently encountered disastrous COP cases. This case illustration and literature review highlight the pathophysiology and management options for DNS after COP. 

## Case presentation

Recently, we came across disastrous COP cases after a fire in a shared accommodation. The hostel was overcrowded and had a faulty air-conditioning/exhaust system. We received a total of five patients. The patient characteristics are given in Table 1.

**Table 1:** Patient characteristics BMI: body mass index, GCS: Glasgow Coma Scale, ECG: electrocardiography ^✱^Troponin T level indicates a degree of myocardial injury. ^✱✱^ Post-resuscitation neurological outcome was said to be good if GCS was more than 8/15, and it was said to be poor if GCS was less than 8/15.

SN	Patient	Case 1	Case 2	Case 3	Case 4	Case 5
1	Age (years)	41	25	34	40	40
2	Sex	Female	Female	Female	Female	Female
3	BMI (kg/m^2^)	31.2	25.8	31.8	28.5	29
4	Carlson comorbidity index	0	0	0	0	0
5	Extraction time from scene to hospital (minutes)	40	60	50	55	60
6	Carboxy hemoglobin on admission (%)	25	18.4	37.4	13.7	19
7	GCS on arrival	03/15	05/15	03/15	03/15	03/15
8	Shock requiring vasopressors	Yes	Yes	Yes	Yes	Yes
9	Serum lactate (mmol/L)	8.1	8.3	9.7	5	10.8
10	ECG	Normal	Normal	Normal	Normal	Normal
11	Troponin T^✱^ (<14 ng/L)	663	446	138	224	1314
12	Neurological outcome^✱✱^	Good	Good	Good	Poor	Poor

Case 1

The patient was a 41-year-old female with no known comorbidities except that her body mass index (BMI) was 31.2 kg/m^2^. She presented to our hospital with altered mental status. Her Glasgow Coma Scale (GCS) score on admission was 3/15. She was in shock. Her relevant labs were a carboxyhemoglobin of 25%, serum lactate of 8.1 mmol/L, and Troponin T of 663 ng/L. She was intubated and mechanically ventilated. Standard ICU management was given to her, which included oxygenation, sedation, fluid resuscitation, and vasopressors. Her carboxyhemoglobin was reversed within an hour after receiving proper oxygenation. She was removed from the ventilator on the second day. Subsequently, she was discharged from the hospital in stable condition on the eighth day.

Case 2

The patient was a 25-year-old female with no known comorbidities. She presented to our hospital with altered mental status. Her GCS score on admission was 5/15. She was in shock. Her relevant labs were a carboxyhemoglobin of 18.4%, serum lactate of 8.2 mmol/L, and Troponin T of 446 ng/L. She was intubated and mechanically ventilated. Standard ICU management was given to her, which included oxygenation, sedation, fluid resuscitation, and vasopressors. Her carboxyhemoglobin reversed within 50 minutes after receiving proper oxygenation. She was removed from the ventilator on the second day. Subsequently, she was discharged from the hospital in stable condition on the seventh day.

Case 3

The patient was a 41-year-old female with no known comorbidities except that her BMI was 31.8 kg/m^2^. She presented to our hospital with altered mental status. Her GCS score on admission was 3/15. She was in shock. Her relevant labs were a carboxyhemoglobin of 37%, serum lactate levels of 9.3 mmol/L, and Troponin T levels of 138 ng/L. She was intubated and mechanically ventilated. Following that, she was given standard ICU care, which included oxygenation, sedation, fluid resuscitation, and vasopressors. After proper oxygenation, her carboxy hemoglobin was reversed within two hours. On the third day, she was removed from the ventilator. Subsequently, she was discharged from the hospital in stable condition on the ninth day.

Case 4

**Figure 1:**
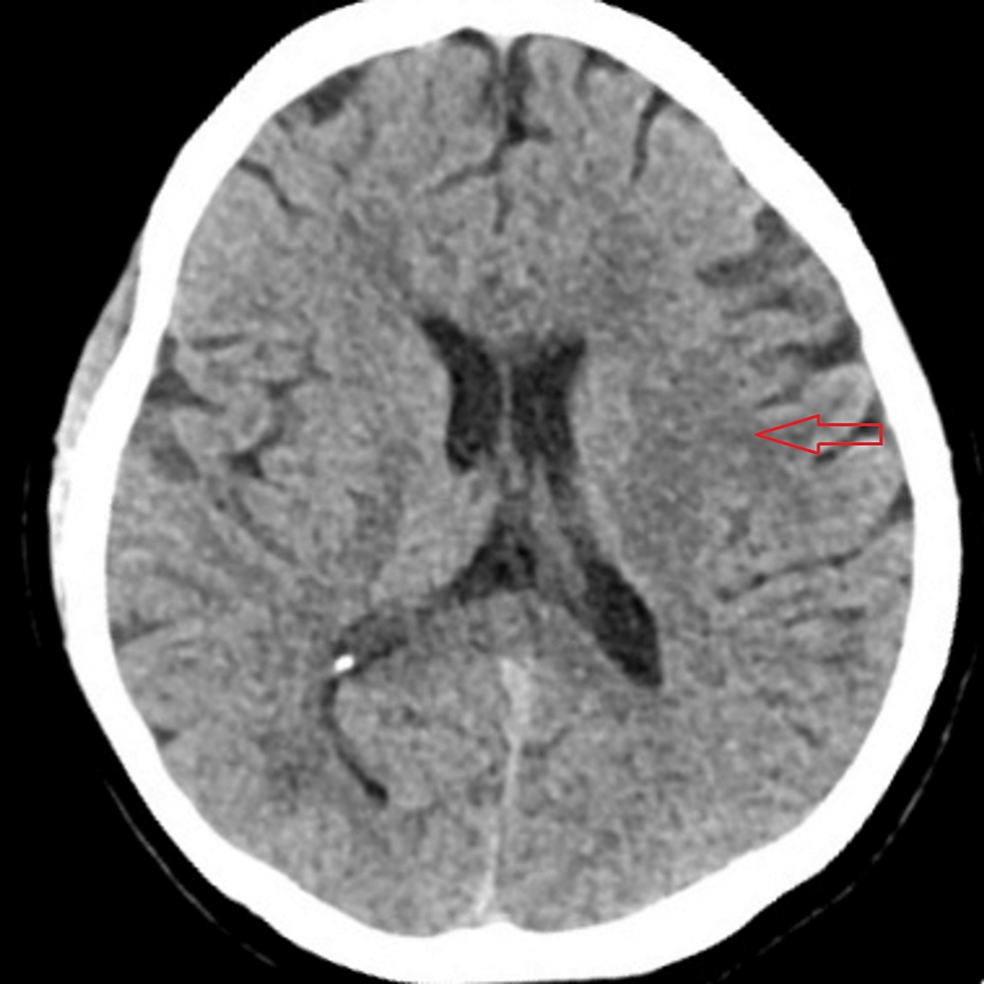
Computed tomography (CT) of the brain of Case 4 CT brain of Case 4 showing a lacunar infarction of the left globus pallidus (red arrow)

The patient was a 34-year-old female with no known comorbidities except that her BMI was 28.5 kg/m^2^. She presented to our hospital with altered mental status. Her GCS score on admission was 3/15. She was in shock. Her relevant labs were a carboxyhemoglobin of 13.7%, serum lactate of 5 mmol/L, and Troponin T of 224 ng/L. She was intubated and mechanically ventilated. Standard ICU management was given to her, which included oxygenation, sedation, fluid resuscitation, and vasopressors. After proper oxygenation, her carboxy hemoglobin was reversed within one hour. We stopped her sedation after stabilizing her condition. But her GCS remains 5/15. Computed tomography of her brain was done, which was suggestive of a lacunar infarct at the left globus pallidus and adjacent genus of the left internal capsule (Figure 1). Depending on her history and neuroimaging, it was clear that she developed DNS after COP. She required a tracheostomy to protect her airway and wean her off the ventilator. After a tracheostomy and allowing her to wean off the ventilator, she was discharged from the ICU.

We kept track of her progress in the general ward. The patient’s GCS remained low like 5T-7T for quite a long time. Her further hospital course was complicated by hospital-acquired pneumonia due to extended-spectrum beta-lactamase-producing Escherichia coli, and she was treated with meropenem 1 gram three times a day for 14 days. Her GCS gradually improved to 11T over 180 days, and she was decannulated on the 188^th^ day. After tracheotomy decannulation, a formal neurological assessment was done on her. She was found to have cognitive decline and exhibited Parkinson's symptoms (tremors, rigidity, hypokinesia, and gait abnormalities). She was treated with supportive measures by a multidisciplinary team consisting of a psychiatrist, neurologist, physiotherapist, and occupational therapist. She showed gradual progress over the next 90 days. Her cognition returned to baseline. Her tremors, hypokinesia, and rigidity resolved with time, but she had a persistent gait abnormality that required walking aids. She was discharged after one month in stable condition with walking aids.

Case 5

**Figure 2:**
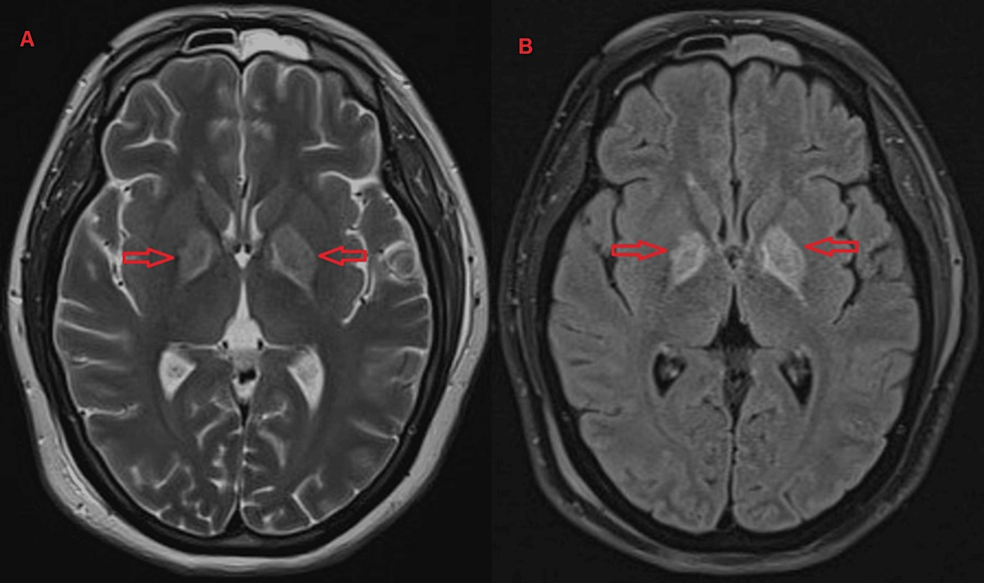
Magnetic resonance imaging (MRI) of the brain of Case 5 MRI brain of Case 5 showing bilateral symmetrical abnormal signal intensity involving the globus pallidi and, to a lesser extent, the centrum semiovale on both sides showing high signal intensity (red arrow) on T2 (2A) and fluid-attenuated inversion recovery (FLAIR) (2B)

The patient was a 41-year-old female with no known comorbidities except that her BMI was 29 kg/m^2^. She presented to our hospital with altered mental status. Her GCS score on admission was 3/15. She was in shock. Her relevant labs were a carboxyhemoglobin of 19%, serum lactate of 10.8 mmol/L, and Troponin T of 1314 ng/L. She was intubated and mechanically ventilated. Standard ICU management was given to her, which included oxygenation, sedation, fluid resuscitation, and vasopressors. After proper oxygenation, her carboxyhemoglobin was reversed within one hour. We stopped her sedation after stabilizing her condition. But her GCS remains 7/15. Magnetic resonance imaging (MRI) of her brain was done which is suggestive of ischemia in globus palladium (Figure 2). Depending on her history and neuroimaging, it was clear that she developed a DNS after COP. She required a tracheostomy for airway protection and weaning from the ventilator. After tracheostomy and weaning from the ventilator, she was discharged from the ICU.

We kept track of her progress in the general ward. The patient’s GCS also remained low at 7T-8T for a substantial period. Her GCS gradually improved after 130 days, and she was decannulated from tracheostomy on the 141^st^ day. After a formal neurological assessment, we noticed that she had a mixed anxiety disorder and distal muscle weakness. She was treated with escitalopram and mirtazapine. Her distal muscle weakness improved with physiotherapy. She was discharged after two months in stable condition without residual neurological symptoms, with antipsychiatry medication and psychiatric follow-up. For both patients (Cases 4 and 5), we consulted an HBO_2_ therapy specialist after the development of DNS. However, because of the rarity of cases, limited evidence, limited experience, and late recognition of DNS, HBO_2_ was not considered in both patients.

## Discussion

Patients with COP can either present themselves with acute COP or with a history of chronic exposure to CO [2,5]. The clinical manifestation of COP depends on the amount and duration of exposure to CO [2,5]. Clinical features of COP are nonspecific, and they may range from mild headache, neuropsychological impairment, confusion, dizziness, ataxia, myocardial/brain infarction, convulsion, and coma, to death [5]. After COP, a noteworthy number of patients may develop a distinct type of neurological dysfunction called DNS [2,4,5].

DNS is a neuropsychological condition noticed after COP, which may have cognitive, psychiatric, vestibulocochlear, motor, sensory, or diffuse demyelinating expression [4,6]. The incidence of DNS is around 45% after COP [4]. Usually, patients present themselves after a lucid interval of 2-40 days [4]. The emergence of DNS is highly unpredictable, and gene regulation or polymorphism may play a vital role in some patients [6]. DNS is usually observed after COP when a victim initially presents themselves with loss of consciousness coupled with hypotension [4]. The pathophysiology of brain injury in DNS is complex. It is caused as a result of hypoxia-anoxia, release of excitatory neurotransmitters, acidosis, ionic imbalance, oxidative stress, nutritive stress, inflammation, and apoptosis [2,4,5,7]. Recently, CO-induced hypoxia of basal ganglia and the subsequent dopamine surge (catecholamine crisis) have been postulated in the pathophysiology of DNS [4]. Pathologically, heme-rich areas of the brain, namely, the third, fourth, and fifth layers of the cortex, hippocampus, basal nucleus, white matter, and Purkinje fiber of cerebellum are mostly affected in DNS. These may show hypoxic-anoxic changes or even demyelination. Clinically, patients after lucid interval may exhibit nonspecific clinical manifestations that include disordered cognition, behavior changes, psychosis, dementia, parkinsonian features, and rarely, severe vestibular or motor deficits [2,4,5]. Usually, Investigation is not required but it is done to rule out other reversible causes of neurological dysfunction. In magnetic resonance imaging (MRI), the brain shows non-specific white matter hypodensities, hippocampal atrophy, or ischemia of metabolically active globous palladium [2,4,5].

Management of DNS after CPO starts with its anticipation. One should anticipate DNS if any patient with COP presents themselves with a loss of consciousness associated with hemodynamic compromise. Treatment is started with the highest possible inspired concentration of normobaric oxygen (NBO_2_), with the main aim being to bring the concentration of carboxyhemoglobin under 5%. HBO_2_ refers to oxygenation at 2.5 to 3 atmospheric pressure. The NBO_2_ decreases the half-life (t1/2) of CO from 320 minutes to 74 minutes, whereas HBO_2_ reduces t ½ of CO to 20 minutes [2,4,5]. HBO_2_ not only causes faster elimination of CO, but it also reverses mitochondrial dysfunction and reduces inflammation [2,4,5]. However, the efficacy of the use of HBO_2_ in preventing DNS gives conflicting results and recommendations in the available medical literature. The American College of Emergency Physicians suggested HBO_2_ in preventing DNS, but they do not recommend its routine use [8]. The Cochrane Database Systemic review done by Buckley et al. does not support the use of HBO_2_ in COP patients to prevent DNS [9]. Another practice recommendation by the American Thoracic Society routinely recommends the use of HBO_2_ in COP victims [10]. In the available medical literature, there is a lack of clarity regarding the optimal atmospheric pressure and the duration and number of sessions of HBO_2_. Some practical/logistic problems related to the institution of HBO_2_ in mechanically ventilated patients, the risk of barotrauma, and decompression sickness associated with HBO_2_ need to be considered. Other potential suggested therapies are therapeutic hypothermia and sympatholytic because hypoxic-anoxia brain injury and catecholamine crisis are the primary pathophysiologies behind DNS development. Hypothermia with sedation may be beneficial in preventing DNS after COP [4]. Some researchers also tried antioxidants (N-acetyl cysteine), pulse steroids (methylprednisolone), or erythropoietin with uncertain benefits [4]. Further, the efficacy of HBO_2_ may be improved, if it is combined with the above-mentioned potential therapy [4]. In prognosis, DNS per se should not account for direct mortality, but these patients might have increased morbidity because of associated critical illnesses. Around 20% and 35% of patients will have cognitive and neurological deficits respectively. Long-term management includes neuropsychiatry consultation and speech, vocational, occupational, and physiotherapy rehabilitation.

## Conclusions

DNS has a high incidence after COP. The DNS is responsible for prolonged hospital stays and increased associated morbidity after COP. Physicians usually encounter COP patients in emergency settings. Our main focus is on maintaining the airway, breathing, circulation, and providing optimal care. The possibility of the development of DNS is often overlooked. So, all primary care physicians should keep in mind this potential sequelae while resuscitating patients with COP or burns. There is still a lack of consensus about the use of HBO_2_ in these patients. Further large-scale prospective studies are needed to know the role of HBO_2_ in COP/DNS. Studies also needed to structure proper guidelines concerning timing, number of sessions, and an optimal atmospheric pressure of HBO_2_ for COP/DNS. Similarly, the other suggested therapy, namely, therapeutic hypothermia, needs further validation by prospective studies. Physicians dealing with COP or burn patients without an HBO_2_ facility should promptly resuscitate all patients with NBO_2 _guided by local protocol and policies. Physicians working in the HBO_2_ facility should, in addition to prompt resuscitation, consider early referral to HBO_2_ therapy.
